# Comparison of Lignocaine-Dexamethasone vs. Lignocaine-Triamcinolone for Preventing Post Spinal-Epidural Backache: A Randomized Study

**DOI:** 10.7759/cureus.75877

**Published:** 2024-12-17

**Authors:** Ankit Soni, Brijesh P Singh, Vinod K Srivastava, Ravi Prakash, Shefali Gautam, Gyan Prakash Singh

**Affiliations:** 1 Anaesthesiology, King George's Medical University, Lucknow, IND

**Keywords:** backache, combined spinal epidural anaesthesia, dexamethasone, lower abdominal surgeries, triamcinolone

## Abstract

Background: The primary objective of this study was to compare the efficacy of lignocaine-dexamethasone and lignocaine-triamcinolone infiltration, along the spinal-epidural needle insertion pathway, to prevent backache after lower abdominal surgeries.

Methods: This prospective, double-blind randomized controlled study included a total of 150 patients, scheduled for elective lower abdominal surgery under combined spinal-epidural (CSE) anaesthesia. The patients were randomised into three groups Group L (Lignocaine, n=50), Group DL (Dexamethasone, Lignocaine, n=50), and Group TL (Triamcinolone, Lignocaine, n=50).

Results: The Visual Analogue Score (VAS) was used to assess the postoperative pain. The anthropometric and demographic findings were comparable among the three groups. The mean VAS score at the time points of needle placement and at postoperative time periods of 24 hours, 48 hours, 72 hours, one month, two months and three months were significantly lower (p<0.001) in groups DL and TL compared to group L.

Conclusion: This study demonstrated that the addition of a steroid like triamcinolone or dexamethasone with lignocaine for local infiltration along the spinal-epidural tract significantly lowers the severity of post-needle puncture backache in comparison to lignocaine alone in lower abdominal surgeries.

## Introduction

The combined spinal-epidural (CSE) anaesthesia is preferably used for lower abdominal surgery, as it offers swift analgesia and continued anaesthesia via subsequent epidural medication administration. The incidence of postsurgical backache after neuraxial anaesthesia is up to 20% [1,2]. The aetiology of postsurgical backache is multifactorial and related to local trauma produced by epidural needle, surgical position and duration, body mass index and the number of attempts to accomplish neuraxial anaesthesia, and among likely causes, neuraxial anaesthesia has been suggested as the potential cause of postsurgical backache [3-6]. It is one of the reasons for patient dissatisfaction and refusal of neuraxial anaesthesia [7-9]. The epidural needle induces local trauma and nerve injury causes pain and edema, and subsequent inflammatory reactions leading to aseptic periostitis, tendonitis, and ligamentous inflammation have been hypothesized as a mechanism of postsurgical back [10-12]. Muslu et al. demonstrated that local administration of nonsteroidal anti-inflammatory drugs before epidural anaesthesia significantly decreased the frequency and severity of post-epidural backache [13]. The efficacy of dexamethasone along with lignocaine for the prevention of post-epidural backache has been well studied [10, 14-16]. Triamcinolone is also a gluco-corticosteroid. It also has anti-inflammatory and immunosuppressive properties by inhibiting phospholipase A2 enzyme, thus inhibiting the biosynthesis of prostaglandin and leukotriene. By combining it with lignocaine in local infiltration in a combined spinal-epidural technique, it also inhibits the synthesis of inflammatory mediators caused by local trauma due to epidural needle, thus preventing the development of acute and chronic backache [17]. The role of triamcinolone has not been studied in the prevention of backache after CSE. The aim of this prospective, double-blinded, randomized controlled study was to compare the effect of lignocaine-dexamethasone and lignocaine-triamcinolone along the spinal-epidural needle pathway to prevent backache in lower abdominal surgeries.

## Materials and methods

This prospective, double-blind, randomised, controlled study was conducted in the Department of Anaesthesiology, King George’s Medical University, Lucknow, over a period of 18 months. The sample size calculated was 150 cases (50 cases in each group) using 90% power and 95% significance level. The sample size is calculated on the basis of incidence of lumbar pain at the first month in the two groups using the formula:

 $n = \left( \frac{z_\alpha \sqrt{2p(1-p)} + z_\beta \sqrt{p_1(1-p_1) + p_2(1-p_2)}}{p_1 - p_2} \right)^2$ 

where p1 = 0.45 (45%), the least sensitivity of the predictor included in the scoring system, p2 = 0.177 (17.7%), the maximum sensitivity of the predictor included in the scoring system [3].

After getting approval from the Institutional Ethics Committee and completing the CTRI registration (registration no. CTRI/2023/08/057040 on August 27, 2023), patients were enrolled in the study. Written and informed consent was taken from the patient.

A total of 150 patients of either sex, aged between 18-50 years, and American Society of Anaesthesiologists (ASA) grade I-II, scheduled to undergo lower abdominal surgeries under CSE anaesthesia were enrolled in this study. Patients were randomised into three groups using computer-generated random number table: Group L received 3 ml Lignocaine 2% + 1 ml normal saline (n=50), Group DL received 1 ml Dexamethasone (4 mg) + 3 ml Lignocaine 2% (n=50) and Group TL received 1 ml Triamcinolone (50 mg) + 3 ml Lignocaine 2% (n=50) for local infiltration along the CSE needle tract before needle insertion.

Patients with allergies to the study drugs or having a general contraindication for CSE anaesthesia, pregnant women and lactating mothers, those having a chronic backache, opioid disuse, psychiatric or neurological disorders, patients who had undergone previous spine surgery and who refused consent were excluded from the study.

The standard ASA monitor (SpO_2_, ECG, non-invasive blood pressure (NIBP), end-tidal carbon dioxide (EtCO_2_), and temperature) was placed for monitoring the patient and intravenous access was secured before the procedure. Patients were placed with a 27-gauge needle (3 cm) either sitting or lateral decubitus and under aseptic precautions, on the skin, subcutaneous tissue, interspinous ligaments, and muscle (spinal-epidural needle path). The study drugs were infiltrated as per the assigned group, 5 minutes prior to CSE placement. The CSE anaesthesia was administered, via 18G epidural needle (Braun Medical (India) Pvt. Ltd., Mumbai) with 27G spinal needle (Braun Medical (India) Pvt. Ltd.) at the level of L2/L3 or L3/L4 via midline approach. Spinal anaesthesia was given with 15 mg bupivacaine heavy and 25 mcg fentanyl followed by epidural catheter placement. Infusion of 0.25% bupivacaine was started @ 5ml/hr via the epidural catheter after 2 hours of spinal anaesthesia and continued for 24 hours. Epidural catheter was removed after 24 hours. The postoperative analgesia was managed with intravenous inj. paracetamol 1 g thrice a day, after the removal of epidural catheter. Inj. diclofenac sodium 1 mg/kg intravenous was used as a rescue analgesic. Postoperative nausea and vomiting (PONV) was managed with intravenous ondansetron 4 mg.

Backache was assessed using VAS during CSE needle placement/insertion, at 24 hours, 48 hours, and 72 hours postoperative. After hospital discharge, the patient was scored for the VAS telephonically at the interval of one week, one month, two months and three months.

Microsoft Excel (Microsoft Corp., Redmond, WA) and IBM SPSS Statistics version 25 (IBM Corp., Armonk, NY) statistical software were used to analyse data. Continuous variables were expressed as mean, while dichotomous variables were represented by number/frequency. Comparisons between groups were made using Student's t-test, Mann-Whitney U test, and Spearman correlation. A significance level of p<0.05 was considered statistically significant.

## Results

The mean age of the groups was comparable, with patients in Group L, DL and TL having a mean age of 41.94±5.8, 42.1±5.6, and 41.8±5.0 years, respectively. Similarly, the mean heights and weights of the groups were comparable, with patients in Group L, DL, and LT having a mean height of 156.6±5.6, 156.5±5.5, and 155.7±5.2 cm, respectively, and having a mean weight of 69.3±9.7, 71.8±8.8, and 69.7±10.3 kg, respectively. The mean body mass index (BMI) for the patients in groups L, DL, and TL was 28.1±3.3 kg/m², 29.3±3.3 kg/m², and 28.7±3.8 kg/m², respectively (Table 1).

**Table 1:** Distribution of the study patients among the three groups based on demographic characteristics and ASA grade ASA: American Society of Anaesthesiologists

Demographic	Group L (n=50)	Group DL (n=50)	Group TL (n=50)	p-value
Gender				
Male	20 (40.0%)	20 (40.0%)	17 (34.0%)	0.775
Female	30 (60.0%)	30 (60.0%)	33 (66.0%)	
Age (years)	41.9±5.8	42.1±5.6	41.8±5.0	0.962
Height (cm)	156.6±5.6	156.5±5.5	155.7±5.2	0.663
Weight (kg)	69.3±9.7	71.8±8.8	69.7±10.3	0.380
BMI (kg/m^2^)	28.1±3.3	29.3±3.3	28.7±3.8	0.229
ASA Grade				
I	5 (10.0%)	9 (18.0%)	19 (38.0%)	0.002
II	45 (90.0%)	41 (82.0%)	31 (62.0%)	

The percentage of patients of groups L, DL, and TL at ASA grade I were 10%, 18% and 38% while those at ASA grade II were 90%, 82% and 62%, respectively. No significant difference in gender distribution was found among the groups.

The mean VAS score at the time points of needle placement and at postoperative time periods of 24 hours, 48 hours, 72 hours, one month, two months and three months were significantly lower (<0.001) in groups DL and TL compared to group L (Table 2).

**Table 2:** Visual Analogue Score (VAS) of the study patients among the three groups

VAS	Groups L (n=50)	Group DL (n=50)	Group TL (n=50)	p-value
At needle placement	2.8±0.73	2.7±0.43	2.1±0.36	<0.001
24 hours	3.6±0.77	3.5±0.73	2.8±0.78	<0.001
48 hours	3.6±0.83	3.4±0.90	2.5±0.78	<0.001
72 hours	3.3±0.91	2.9±0.80	2.4±0.83	<0.001
1 month	2.8±0.71	2.7±0.63	1.9±0.65	<0.001
2 months	2.7±0.70	2.3±0.66	1.8±0.58	<0.001
3 months	2.4±0.67	2.1±0.62	1.6±0.51	<0.001

In the "needle placement" category, the mean VAS scores were 2.8±0.73 for Group L, 2.7±0.43 for Group DL, and 2.1±0.39 for Group TL. The p-value for the comparison between Group L and Group DL was 0.406, indicating no significant difference. However, both comparisons between Group L and Group TL, and between Group DL and Group TL, yield p-values of less than 0.001, indicating significant differences (Table 3).

**Table 3:** Intergroup comparison of Visual Analogue Score (VAS)

VAS	Groups Lb(n=50)	Group DL (n=50)	Group TL (n=50)	p-value group (L vs DL)	p-value Group (L vs TL)	p-value Group (DL vs TL)
Needle placement	2.8±0.73	2.7±0.43	2.1±0.36	0.406	<0.001	<0.001
24 hours	3.6±0.77	3.5±0.73	2.8±0.78	0.596	<0.001	<0.001
48 hours	3.6±0.83	3.4±0.90	2.5±0.78	0.303	<0.001	<0.001
72 hours	3.3±0.91	2.9±0.80	2.4±0.83	0.030	<0.001	<0.001
1 month	2.8±0.71	2.7±0.63	1.9±0.65	0.556	<0.001	<0.001
2 months	2.7±0.70	2.3±0.66	1.8±0.58	0.15	<0.001	<0.001
3 months	2.4±0.67	2.1±0.62	1.6±0.51	0.049	<0.001	<0.001

## Discussion

Epidural anaesthesia is a common technique used in medical settings for anaesthesia, analgesia, and managing chronic pain [10]. Dexamethasone, when combined with local anaesthetics, has been shown to prolong block duration. The exact mechanism behind this prolongation is unclear, but some researchers suggest it may be due to the vasoconstrictive properties of corticosteroids [11,12].

Muslu et al. [13] found that steroidal anti-inflammatory drugs, such as dexamethasone, can relieve post-epidural low back pain. Epidural dexamethasone injections have shown efficacy in alleviating back pain [14,15]. A similar study done on 150 patients also revealed similar demographic data. The mean age of the patients was comparable across the groups, with Group L having a mean age of 41.94±5.8 years, Group DL having a mean age of 42.1±5.6 years, and Group TL having a mean age of 41.8±5.0 years. The mean body mass index (BMI) for the groups was 28.1±3.3, 29.3±3.3, and 28.7±3.8 kg/m², respectively. The mean weight and height of the patients were 62.5±8.8 kg and 161.8±5.9 cm in Group L and 63.4±7.6 kg and 161.4±4.8 cm in Group DL. Similarly in another study, Group A (1.5% lidocaine with dexamethasone) had a mean age of 49.0±8.4 years, while in Group B (1.5% lidocaine with dexamethasone), it was 49.0±7.0 years. In Group L, 20 (40.0%) were male patients, 30 (60.0%) were female patients, and in Group DL, 20 (40.0%) were male patients, 30 (60.0%) were female patients, and in Group TL, 17 (34.0%) were male patients and 33 (66.0%) female patients. There was no significant difference in gender distribution among the groups [3,16].

The study found that Group TL had the lowest mean VAS score at the 24-hour mark post-surgery, followed by Group DL and Group L. This trend persisted at 48 hours, 72 hours, and across subsequent months, with Group TL consistently showing the lowest VAS scores. There were significant differences (p<0.001) in VAS scores among the three groups at all time points. The intragroup VAS scores were 2.8±0.73 for Group L, 2.7±0.43 for Group DL, and 2.1±0.39 for Group TL. The p-value for the comparison between Group L and Group DL was 0.406, indicating no significant difference. However, both comparisons yielded p-values of less than 0.001, indicating significant differences. These findings were in line with a study by Pahuja et al., which reported very low pain scores (69.56%) [17].

Epidural dexamethasone may reduce postoperative pain after gynaecological surgeries by reducing the occurrence and severity of back pain. Studies have shown that local infiltration of dexamethasone along the spinal-epidural tract leads to lower back pain after surgery. The consumption of fentanyl for back pain management was also documented. The Dexamethasone-Lignocaine (DL) group showed lower fentanyl usage during the initial three postoperative days compared to the Lignocaine (L) group. Moreover, among patients without preoperative lumbago, the DL group significantly reduced back pain occurrence during the first, second, and sixth months. The incorporation of dexamethasone into local lidocaine infiltration effectively mitigates both the frequency and intensity of back pain after combined spinal-epidural anaesthesia. Injections of triamcinolone acetonide in small doses with lignocaine were more successful in treating chronic backache than lignocaine alone. Combining steroidal anti-inflammatory drugs with lidocaine locally could decrease the occurrence and intensity of post-epidural backache compared to lidocaine alone [3,5,18-20].

A randomised control study done by Tagowski et al. on patients with lumbar radiculopathy found that pain relief, baseline pain intensity and the presence of a herniated intervertebral disc were significant predictors of achieving ≥50% pain relief in lumbar epidural dexamethasone injection [21]. Patients with a baseline Numeric Rating Scale (NRS) score ≥7 had a lower likelihood of experiencing ≥50% pain relief compared to those with an NRS score <7. However, the presence of disc herniation more than doubled the chances of achieving ≥50% pain relief. Triamcinolone exhibited superiority in managing severe lumbar radiculopathy, but no discernible advantage was observed in mild to moderate pain [22]. The effectiveness of dexamethasone was comparatively lower for stenotic spinal lesions than for disc herniation. Pain relief was evident by three weeks, with no discernible difference between the groups [22]. Both the methylprednisolone and triamcinolone groups demonstrated greater improvement. Overall, pain relief was markedly superior at all follow-up assessments in the steroid group compared to the control group.

In our study, we found that the combination of triamcinolone acetonide and lignocaine for local infiltration is highly effective in preventing backache compared to lignocaine alone. The limitations of this study include a small sample size and lack of long follow-up duration.

## Conclusions

Spinal-epidural needle puncture can lead to acute and chronic backache, which can be very disturbing for the patient. The incidence of postoperative backache after spinal-epidural needle puncture can be effectively reduced with the addition of steroids to local anaesthetic. Triamcinolone, when added to lignocaine used for local infiltration along the needle tract, can reduce backache compared to the local anaesthetic lignocaine alone. Triamcinolone is better than dexamethasone in reducing backache when added to lignocaine for infiltration along the needle tract. Therefore, we recommend adding triamcinolone to lignocaine for local infiltration along the spinal-epidural needle tract.
